# Effectiveness of Life Skills Intervention on Depression, Anxiety and Stress among Children and Adolescents: A Systematic Review

**DOI:** 10.21315/mjms2023.30.3.4

**Published:** 2023-06-27

**Authors:** Yosra Sherif, Ahmad Zaid Fattah Azman, Hamidin Awang, Siti Aisha Mokhtar, Marjan Mohammadzadeh, Aisha Siddiqah Alimuddin

**Affiliations:** 1 Department of Community Health, Faculty of Medicine and Health Sciences, Universiti Putra Malaysia, Selangor, Malaysia; 2 Psychiatry Unit, Faculty of Medicine and Health Sciences, Universiti Sains Islam Malaysia, Negeri Sembilan, Malaysia; 3 Institute of Health and Nursing Sciences, Charité-Universitätsmedizin Berlin, Corporate Member of Freie Universität Berlin and Humboldt-Universität zu Berlin, Berlin, Germany; 4 Department of Psychiatry, Faculty of Medicine and Health Sciences, Universiti Putra Malaysia, Selangor, Malaysia

**Keywords:** mental health, depression, anxiety, stress, children, adolescent, life skills

## Abstract

Children and adolescents are at a significantly high risk of mental health problems during their lifetime, among which are depression and anxiety, which are the most common. Life skills education is one of the intervention programmes designed to improve mental well-being and strengthen their ability to cope with the daily stresses of life. This review aimed to identify and evaluate the effect of life skills intervention on the reduction of depression, anxiety and stress among children and adolescents. Following the Population, Intervention, Comparison and Outcome (PICO) model and the Preferred Reporting Items for Systematic Reviews and Meta-analyses (PRISMA) 2009 checklist, eight databases (Academic Search Complete, CINAHL, Cochrane, MEDLINE, Psychology and Behavioural Sciences Collection, PubMed, Scopus and Web of Science) were systematically reviewed from 2012 to 2020. The search was limited to English papers only. It included published experimental and quasi-experimental studies addressing the effect of life skills interventions on the reduction of at least one of the following mental health disorders: depression, anxiety and stress among children and adolescents (from the age of 5 years old to 18 years old). We used the Joanna Briggs Institute checklist for experimental and quasi-experimental studies to evaluate the quality of the included studies. This study was registered in PROSPERO [CRD42021256603]. The search identified only 10 studies (three experimental and seven quasi-experimental) from 2,160 articles. The number of the participants was 6,714 aged between 10 years old and 19 years old. Three studies in this review focused on depression and anxiety, whereas one study investigated depression and the other anxiety. Three studies targeted only stress and two examined the three outcomes, namely, depression, anxiety and stress. Almost in all studies, the life skills intervention positively impacted mental disorders, considering the differences among males and females. The overall methodological quality of the findings was deemed to be moderate to high. Our results clearly indicated the advantages of life skills programmes among adolescents in different settings and contexts. Nonetheless, the results highlight some important policy implications by emphasising the crucial roles of developers and policymakers in the implementation of appropriate modules and activities. Further research examining life skills intervention with a cultural, gender perspective, age-appropriate and long-term effect is recommended.

## Introduction

One of the growing public health issues among children and adolescents is mental disorders (1), which is recognised as a priority topic for more research and government intervention (2). It is estimated that 10% to 20% of children and adolescents globally have experienced mental health problems. Furthermore, a more significant proportion of mental health problems has been observed for specific subgroups of teenagers, those with socioeconomically disadvantaged positions and those who lack appropriate health or social services, are identified as minority ethnic groups and live in more rural or distant locations (2–4).

In Europe and the USA, mental illnesses account for most disability-adjusted life years among children between 5 years old and 14 years old (5). The findings from previous research indicated that anxiety and depression are common among children aged 8 years old–12 years old, with reported prevalence rates of approximately 2% and 5%, respectively (6). Moreover, adolescence is a sensitive and crucial stage for development from childhood to adulthood (7). Multiple physical, emotional and social changes occur during this formative time of adolescence (8). These changes can make adolescents vulnerable to mental health problems and nearly half of these problems begin before the age of 14 (9). Mental health conditions account for 16% of the global burden of disease and injury among adolescents (8). Furthermore, these problems have been demonstrated to increase the risk of adverse consequences, such as impairment, lack of productivity and ability to contribute to society, low educational performance and increased probability of exhibiting risky behaviours, such as alcoholism and sexual, and suicidal behaviours (10).

Anxiety and depression are the most prevalent mental health conditions during the early life (11). These disorders commonly emerge during childhood and adolescence but might continue until adulthood if left untreated. Depression and anxiety are the 4th and 9th leading causes of illness and disability in late-stage youth and the 15th and 6th in early-stage adolescents, respectively (12). Moreover, these disorders could have a long-term and repeating effect and are more likely to co-occur together up to 50% (4, 11). Depression is the primary cause of disability-adjusted life years lost in teenagers worldwide. It occurs in 2%–8% of children and adolescents, with the highest prevalence during puberty. Of the affected individuals, around 40% experience repeated episodes and approximately 33% think about suicide, with 3% to 4% actually committing it (13, 14).

Meanwhile, 1 in 10 young individuals suffers from anxiety disorders before reaching the age of 16 (15). According to the World Health Organization (WHO), the prevalence of this disorder was between 5.7% and 17.7% in children and adolescents (16). Similarly, stress is a mental health condition that negatively impacts people’s lives. During adolescence, the susceptibility to stress is highly increased, adversely affecting individuals’ psychological and physical well-being (17).

Prevention is one strategy to reduce the burden of these illnesses, which can be categorised as either universal or targeted programmes (11). It is necessary to address these disorders by implementing educational programmes targeting diverse children and teenagers to introduce and reinforce essential knowledge and skills in mental well-being (18). School is a suitable atmosphere for targeting adolescents. It demonstrates the most effective social settings that can help students practice cognitive and social skills as they spend a significant amount of their time there. Furthermore, it offers intervention opportunities with the support of social relationships. School-based mental health programmes can reduce and alleviate many common barriers to treatment in the community, such as cost, location, time, transportation and stigmatisation, by offering alternatives that are of low cost, have high utilisation levels, are convenient and non-threatening (19, 11, 20, 21). School plays a vital role in identifying those with symptomatic and those at risk of becoming symptomatic (11).

**Figure 1:**
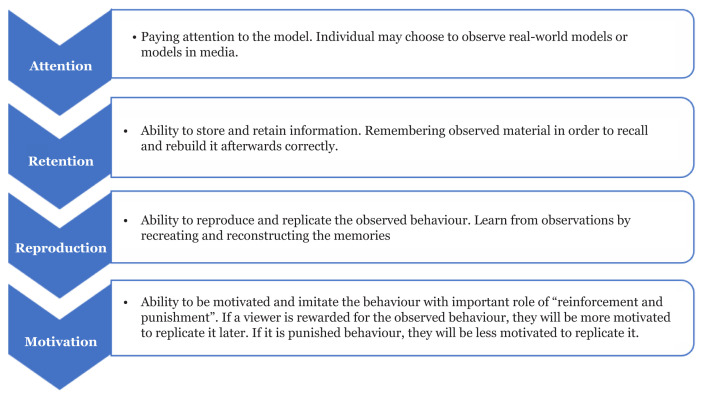
Schematic outline of observational learning and modelling process in social learning theory *Source*: Nabavi (27)

Life skills education is an organised educational programme designed to improve children and adolescents’ skills and abilities, enabling them to deal more effectively with the daily demands of life (22, 23). It also aims to improve mental health and boost the positive and adaptive behaviours of the target individuals (24). According to the WHO, life skills are generally defined as ‘abilities for adaptive and positive behaviour that enables individuals to deal effectively with the demands and challenges of everyday life’ (25). The theoretical foundation of the life skills programme is based on the social learning theory developed by Albert Bandura in 1977 (24, 25). He stated that people learn through observational learning, imitation and modelling. Bandura introduced the term ‘observational learning’ and defined the components of appropriate observational learning as attention, retention, reproduction and motivation (Figure 1).

He posited that individuals observe and copy the behaviour of others in their social worlds and develop an idea of how new actions are performed. This recorded information serves as a guide for action on subsequent occasions. His explanation on observational learning enables an individual to rapidly gather knowledge by observing and imitating models found in his/her environment. Then, in 1986, Bandura highlighted the cognitive aspects of observational learning, and manner, behaviour, cognition and environment interact to shape individuals. He introduced the principle of the dynamic and reciprocal relationship between a person (an individual with a collection of previous experiences), their environment (the external social circumstances) and their behaviour (responses to stimuli to achieve goals) (26–28).

Life skills education includes activities that support critical and creative thinking, coping with emotions and stress, self-awareness and empathy, decision-making and problem-solving, communication skills and interpersonal relations (25). Life skills education has been used in different countries and targets different health outcomes, such as improvement and promotion of mental (25, 29), psychosocial (30), and physical health and prevention of acquired immunodeficiency syndrome AIDS (31), substance abuse (32) and teenage pregnancy (22, 33). Thus, life skills education has been established for preventive measures, promoting healthy positive behaviour, and strengthening communication and socialisation skills.

Therefore, this systematic review aimed to provide an overview and summarise the available literature about the effect of life skills programmes on the reduction of depression, anxiety and stress among children and adolescents. In addition, it would provide good insight into the appropriate approach for implementing the accurate methods. Following the PICO model, the main review question of the current systematic review is as follows: What is the effect of life skills intervention on depression, anxiety and stress levels among children and adolescents (5 years old–18 years old of age)?

## Methods

The current systematic review and the bibliometric study were conducted by following the ‘PRISMA’ 2009 checklist (34). The protocol of this systematic review was registered in the International Prospective Register of Systematic Reviews (PROSPERO) (registration number: CRD42021256603).

### Literature Search and Eligibility Criteria

A comprehensive search was initially conducted on eight electronic databases, namely, Academic Search Complete, CINAHL, Cochrane Library, MEDLINE, Psychology and Behavioural Sciences Collection, PubMed, Scopus and Web of Science. The following keywords were used in the search: Population: (Children OR child OR adolescents OR youth OR young OR teen OR teenage OR young people OR young adult), Intervention: AND (‘life skills’), Outcome: AND (‘mental disorders’ OR ‘mental health’ OR ‘internalising problems’ OR ‘emotional problems’ OR ‘anxiety disorders’ OR ‘depressive disorders’ OR ‘depression’ OR ‘stress’ OR ‘anxiety’ OR ‘Psychological stress’ OR ‘Life Stress’ OR ‘emotional stress’). Only databases from 2012 to 2020 were searched. The literature was limited to the English language due to the expected translation problem. The detailed search strategy of the electronic databases is illustrated in the supplementary table (Table S1).

The inclusion criteria were as follows: i) participants were children or adolescents with ages between 5 years old and 18 years old; ii) intervention was the life skills programme; iii) life skills intervention groups compared with either school-as usual control groups, waitlist control groups or other educational interventions or no control groups; iv) the studies reported at least one mental health outcome, either depression, anxiety and stress, at baseline and post-intervention at a minimum; v) randomised controlled trials (RCTs) and non-randomised controlled trials (non-RCT), such as quasi-experimental and pre-post studies design. Studies were excluded if: i) the studies evaluated drug and alcohol use, physical and sexual activities, and nutritional interventions; ii) non-English studies and iii) non-experimental studies, such as observational (e.g. cross-sectional, case-control and cohort studies) and qualitative ones.

### Data Collection and Analysis

All citations were uploaded into the Mendeley software and duplicated studies were removed. Two reviewers screened the titles, abstracts, and, finally, full texts based on the inclusion criteria. Disagreements were resolved through a discussion between the two reviewers. If the disagreement remained, a third person was available to arbitrate.

### Data Extraction and Management

Two reviewers independently collected the standardised data extraction forms. The information extracted included the following: first author, year of publication, country, study design (RCT or non-RCT), participant’s age, sample size, instrument, intervention characteristics and findings.

### Quality Appraisal

Two independent reviewers used the Critical Appraisal Checklist for RCTs and Quasi-Experimental studies developed by the Joanna Briggs Institute (JBI) (35) to evaluate the risk of bias for the eligible studies. In addition, they calculated the overall risk score based on the number of items checked for each evaluation. The purpose of this appraisal was to assess the methodological quality and determine the possibility of bias in the study design, conduct and analysis. Any disagreement between the reviewers was addressed by discussion.

The instruments consisted of 13 and 9 questions for the RCTs checklist and the quasi-experimental checklist, respectively. These questions were answerable by ‘yes,’ ‘no,’ ‘unclear,’ or ‘not applicable.’ The appraisal score represented the percentage of (yes) responses from the total number of questions. At least 50% of the ‘yes’ scores on the JBI critical evaluation instruments were used as the cut-off point for inclusion in the RCT and quasi-experimental trial review (36). When a criterion was ‘not reported,’ it was considered as ‘unclear’ and treated as a ‘no’ response. If a measure did not apply (N/A) to the study, that item was not counted in the total number of criteria (37).

## Results

### Study Selection

**Figure 2:**
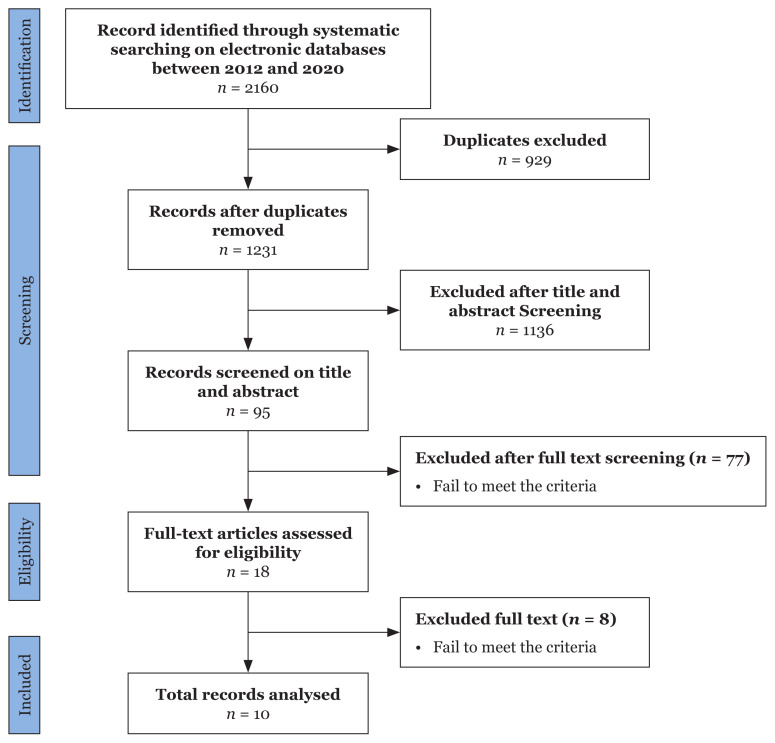
Prisma flow diagram of the selected articles

According to the PRISMA diagram (Figure 2), a total of 2,160 articles were identified in the initial database search. After removing the duplicates, 1,231 articles were further examined, of which 1,136 were excluded during the title and abstract screening. A total of 18 full-text articles were left for eligibility assessment. Finally, 10 articles were found to meet the eligibility criteria.

### Study Characteristics

Detailed information about the authors, year of publication, countries, study design, sample size, participants, instrument, intervention characteristics, findings and summary of the results is provided in Table 1. The studies included in the review were seven quasi-experimental ones and three were RCTs. All the included studies were conducted in seven different countries: one study in Malaysia (29); one study in Taiwan (38); three studies in Iran (39–41); two studies in India (42, 43); one study in Uganda (44); one study in Kenya (45) and one study in Australia (46).

### Participants’ Characteristics

The total number of participants in the included studies was 6,714 with varying sample sizes from 40 adolescents in Australia (46) to 2,522 in Taiwan (38). Most studies recruited individuals from schools, one in Malaysia from orphanages (29) and one in Iran from paediatric hospitals (40). The age range in all studies was 10 years old–19 years old; however, no research was conducted among children. Both gender, males and females, participated in most studies, only one study was conducted among boys (43) and another one among girls (41).

### Intervention Characteristics

All studies in this review investigated the effect of life skills intervention on the reduction of depression, anxiety and stress among adolescents (Table 2). Three studies targeted only stress (39, 42, 43), and one study each targeted depression (38) and anxiety (46). Meanwhile, three other studies focused on depression and anxiety-like symptoms (40, 44, 45), and the last two targeted three mental conditions, namely, depression, anxiety and stress, altogether (29, 41). Baseline assessment was performed for all the participants and the findings were compared with the post-intervention results, except for one study, which did not include pre-intervention evaluation (38). The period of post-intervention assessment differed in the included studies, ranging from immediately after the intervention to 9 months (45) and 1 year (43). In a study by Lee et al. (38), there were no follow-ups, only post-test assessments. Detailed information and a summary of the intervention assessment and follow-up are presented in Table 2. Four studies evaluated the effect of the intervention by comparing the intervention and control groups; only one study had no control group (45). The intervention programme was conducted by the researcher in most of the included studies.

Meanwhile, the trained teachers conducted the programme in other studies and only one study was performed by a clinical psychologist (40). The length and contents of the intervention were also different from one study to another. The overall duration of the intervention ranged from 1 week to months and the length for each session ranged from 45 min to 150 min. The education modules were slightly different among the included studies. They used various activities such as brainstorming, goal-setting, role-playing and group discussion, drama, drawing, playing games and matches, and question-and-answer sessions.

### Quality Appraisal

As presented in Table 1, the appraisal score for the methodological quality (in percentage) of the included studies ranged from moderate (62%) to high (100%), where high quality was regarded as more than 80%, moderate quality as 50% to 79% and poor quality as less than 50% (37). Half of the studies were of moderate quality, whereas the rest were considered to be of high quality. The comprehensive data on the methodological quality of the included studies are presented in the supplementary tables (Tables S2 and S3).

## Discussion

In this systematic review, we identified and summarised the effect of life skills intervention on depression and/or anxiety and/or stress among children and adolescents. The study demonstrated that the life skills intervention positively influenced the adolescents’ mental health. It also provided evidence supporting the development and establishment of life skills interventions. Our findings are consistent with those of previous research, indicating the efficiency and effectiveness of educational programmes and mental health interventions (18, 29, 11, 47–49).

Several aspects of the effect of life skills programmes are highlighted in this review. For instance, the life skills intervention is based on three critical key elements, namely, appropriate educational strategies, active educational techniques and safe learning environments. Furthermore, the link between theoretical and practical aspects is one of the essential educational strategies. Four articles in this review mentioned the life skills intervention-based theories: stress-coping theory (29), social cognitive theory (45) and self-determinant theory (46). The teaching and learning approaches are situated at the junction of the conceptual and programmatic frameworks for life skills. Life skills education is also focused on two main aspects. First, life skills are changeable; they are not permanent character traits and may thus be taught, learned and acquired throughout life. Second, they can be reinforced through proper educational interventions. In this sense, because teaching and learning are integral components of life skills, a fundamental practical aspect of life skills programmes is the determination of the most effective teaching and learning methods.

In addition, active learning is the most effective method for delivering life skills education. It includes a learner-focused approach that places importance on the teaching and learning process. Active learning methods encourage students to become active participants in their education rather than becoming passive information users. Students are considered as active thinkers who may be stimulated by engaging in instructional approaches. They work with other students to improve their talents and, as a result, form strong bonds with their classmates. It is also critical to consider children and youth’s perspectives, ideas, and concerns while assuring their active involvement in educational activities. Another vital component of participatory education, small group and teamwork have several benefits for successful life skills education.

More insight into the importance of schools as safe environments can contribute to the success of life skills intervention and can help create an excellent ground for teachers and peer relationships. Schools are ideal environments for interventional and training studies on children and adolescents because of easy access to many participants, a high degree of confidence among parents and the community, and the possibility to evaluate the short- and long-term impact of the studies (11, 19–21). Consequently, incorporating life skills training as part of the school curriculum at early stages is also necessary. It can facilitate the early recognition of students experiencing problems with their emotional health and well-being as well as the referral to appropriate support.

Our results contributed to this field of study by emphasising the critical components of success among teenagers that reflect numerous aspects of other mental health outcomes. Life skills interventions promote positive mental health and encourage teenagers with essential skills to improve their abilities and overcome challenges (50). Moreover, it plays a significant role in enhancing students’ success in both academic and non-academic areas (40), such as strengthening of coping mechanisms (29) and development of self-confidence (30, 40) and empathy (51). Accordingly, good mental health and well-being influence healthy behaviours, improved physical health, high educational achievement, high productivity, jobs and income. Eventually, teenagers show positive changes from the knowledge gained about the different coping strategies and life skills (38, 52).

Although the duration of the life skills programmes appeared different in this review, its effect on the studied variables was achieved. The priority was focused on the intensity of the sessions and the quality of the presented material, instead of the number of sessions. Most of the included studies were limited to the documentation of short-term effects obtained through methodologies with small sample sizes; in addition, they were restricted to pre-post-test assessments, without any follow-up, to fully evaluate the effectiveness of the respective activities. These findings indicate the need for additional research to fully evaluate the respective programme’s performance. Furthermore, long-term monitoring and assessment are required to construct empirical evidence with regard to the success of life skills interventions (6). Regular booster sessions and reinforcement must also be considered for the maintenance of mental health well-being.

Thus, the implementation of sustainable life skills programmes is a crucial element. As a result, greater focus is needed in these situations on the establishment of continuous and sustainable programmes through systematic planning, organisation, supervision and assessment of teaching these skills (22, 53). The use of an appropriate instrument for the assessment of outcomes can help in the production of high-quality results. Although various tools have been used to evaluate and measure depression, anxiety and stress, they are suitable for children and adolescents. The validity and reliability of the rating scales were documented in most of the included studies, validating the quality of the studies.

Life skills programme mentors, policymakers, officials and instructors must understand its potential and worth and receive adequate training (19, 54, 55). In this setting, increasing access to appropriate interventions is necessary, especially those provided by non-healthcare professionals, such as teachers and caregivers.

Considering adolescent experiences in the context of an individual’s tradition and culture is crucial for comprehending how individuals from varied backgrounds acquire life skills knowledge. This may include student comments and discussions on each life skills issue to enhance the applicability of the skills (24, 25). Studies might be described in terms of experiences, such as stories from teenagers’ lives, examination of different perspectives and the distinct social circumstances in which they acquire life skills. These perspectives will create a more balanced understanding of the realities of programme effectiveness.

Furthermore, it can be noticed that in the included studies, life skills education has been integrated into particular social and cultural contexts. For instance, in the Malaysian research (29), the programme was conducted in orphanages in the Malay language and targeted the Malaysian environment and local culture, with respect to ethnicity and religion. Meanwhile, in Taiwanese schools (38), the curriculum was translated to Mandarin’s local language. It was modified and changed using Taiwanese life-experience situations to ensure that it was known to Taiwanese students and practical in a school and classroom setting. Moreover, they used the most conventional social media application in Taiwan for further discussion.

In the Tibetan study (42), the programme was also constructed for Tibetan refugee teenagers by making the life skills activities realistic and relevant to the refugee experience. The names of characters and locations have been changed and replaced to reflect the situation of Tibetan refugee youths. In Uganda (44), Kenya (45), Australia (46) and Iran (40), the activities were designed based on the particular psychological needs of the students’ skills. The objective was to create a curriculum suited for those participants’ cultural and social contexts. Here, it can be noticed that the basic concept of life skills intervention is the same across different countries. Moreover, these skills were contextualised according to the social and cultural context and settings. Therefore, certified trainers who customise the curriculum with more appropriate examples and real-life situations closer to the user’s background would make the life skills programme more effective and impactful.

Another important finding in our review is the lack of life skills intervention studies among children due to several reasons. First, the prevalence of mental disorders peaks during adolescence. It is a transitional stage characterised by rapid growth and development with the occurrence of numerous physical and psychological changes, such as increased susceptibility to stressors and the emergence of many mental health disorders (14, 17, 56, 57). Also, previous literature documented that the symptoms of these mental disorders persist throughout childhood; thus, it is not common for intervention programmes to focus on children (6). Furthermore, the limited search on the database might lead to missing relevant articles before 2012 and those in non-English languages. Finally, we excluded different study designs that target children, such as the mixed-method design.

Gender disparity in the interpretation of mental illness is reported in this review. For example, symptoms of depression were lesser in males but not in females after the life skills intervention. Similarly, previous literature documented the presence of gender inequality in adolescents who experienced internalising and externalising problems. Females tended to have higher levels of internalising problems, whereas males tended to have higher levels of externalising problems (58). Females generally showed more emotional reactions to stressful situations, whereas males exhibited more cognitive responses (59, 49). This could mean that females are more susceptible to the risk factors owing to their biological differences (29).

Meanwhile, males were observed to practice more skills than females and regulate their emotional symptoms better than females. In addition, females used social support as a coping method, even though it has been observed that females who sought social support were more likely to experience mental health issues, but not males (60). Consequently, more advanced research focusing on gender variation and how various life skills interventions impact these populations is needed. Such an effort could help promote dedicated sections where males and females could be separately addressed.

Addressing the concerns and challenges faced by children and young adults in early life through education programmes could make them independent in coping with life’s demands, which can transform these challenges and obstacles into opportunities. In addition, the cultural and sustainable development of the programme is crucial, which involves indigenous individuals as consultants and local assistants in policymaking. It will contribute to sociocultural awareness, decreasing the possibility of inappropriate implementation.

Although a comprehensive search using eight databases was performed to obtain an enormous number of studies, our systematic review has several limitations. Our search was limited to articles published between 2012 and 2020. Furthermore, it did not include non-English articles and gray literature; thus, it is possible that some relevant studies have been missed. Furthermore, some of the studies that involved multicomponent interventions were not included, making narrative synthesis and interpretation of the evidence challenging. Lastly, the differences in the study population, location, sample size, study length and instruments across the included studies make it difficult to effectively compare the intervention.

## Conclusion

This review has synthesised evidence on life skills intervention to improve the mental health of adolescents. It identifies several experimental and quasi-experimental studies that evaluated life skills programmes as a potential intervention strategy for effectively addressing teenagers’ mental well-being through the reduction of depression, anxiety and stress. The methods used by adolescents to acquire information and skills through life skills programmes and then to adopt good attitudes and behaviours were explained in almost all studies.

Life skills education was focused on specific life skills, depending on the setting. It considered psychosocial competencies and interpersonal skills that help participants in making the right decisions, solving problems, thinking critically and creatively, communicating effectively, building healthy relationships, empathising with others, and coping with managing their lives in a healthy and productive manner.

The current research has resulted in numerous critical recommendations on the development of life skills educational interventions. First, life skills development is at the core of childhood and adolescence protection strategies. Future research is recommended to holistically examine life skills educational intervention to provide robust evidence on its effectiveness and to achieve long-term effects.

In addition, a comprehensive approach with a cultural, gender perspective, age-appropriate and active learning should be considered. Based on the evidence in this review, policymakers, officials and health professionals are suggested to offer life skills training programmes to all children and adolescents in schools and institutions. In addition, it could benefit from providing resources using internet applications to enable fast and easy access to information.

## Supplementary 1

Search strategy for databases

- The electronic databases were initially searched.
- They were Academic Search Complete, CINAHL, Cochrane, MEDLINE, Psychology and Behavioural Sciences Collection, PubMed, Scopus and Web of Science.

The title and abstract of articles searched using several keywords are as follows:

- Population: (Children OR child OR adolescents OR youth OR young OR teen OR teenage OR young people OR young adult),
- Intervention: AND (‘life skills’),
- Outcome: AND (‘mental disorders’ OR ‘mental health’ OR ‘internalising problems’ OR ‘emotional problems’ OR ‘anxiety disorders’ OR ‘depressive disorders’ OR ‘depression’ OR ‘stress’ OR ‘anxiety’ OR ‘Psychological stress’ OR ‘Life Stress’ OR ‘emotional stress’).

The literature was limited to the English language because of the expected translation problem.

**Table S1:** Electronic databases

Database	Number
Academic Search Complete	591
MEDLINE Complete	357
CINAHL Plus with full text	208
Psychology and Behavioural Sciences Collection	163
Cochrane Central Register of Controlled Trials	66
Cochrane Database of Systematic Reviews	3
Web of Science	218
Scopus	400
PubMed	154
All	2160
Narrow by Langue	English

## Supplementary 2

**Table S2:** Methodological quality of randomised controlled trial

Studies	Criteria													

	1*	2*	3*	4*	5*	6*	7*	8*	9*	10*	11*	12*	13*	Overall
Mohammadzadeh et al. ( 29)	Y	Y	Y	Y	N	N	Y	Y	Y	Y	Y	Y	Y	11/13 85%
Lee et al. ( 38)	Y	Y	Y	U	N	U	Y	U	N	Y	Y	Y	N	8/13 62%
Jamali et al. ( 39)	Y	N	Y	U	U	U	Y	Y	Y	Y	Y	Y	Y	9/13 69%

Total	3/3 100%	2/3 66%	3/3 100%	1/3 33%	0/3 0%	0/3 0%	3/3 100%	2/3 66%	2/3 66%	3/3 100%	3/3 100%	3/3 100%	2/3 66%	

Notes: JBI methodological quality appraisal checklist to be scored as: Yes = Y; No = N; Unclear = U; Not applicable = NA;

1* Was true randomisation used for assignment of participants to treatment groups?

2* Was allocation to treatment groups concealed?

3* Were treatment groups similar at the baseline?

4* Were participants blind to treatment assignment?

5* Were those delivering treatment blind to treatment assignment?

6* Were outcomes assessors blind to treatment assignment?

7* Were treatment groups treated identically other than the intervention of interest?

8* Was follow up complete and if not, were differences between groups in terms of their follow up adequately described and analysed?

9* Were participants analysed in the groups to which they were randomised?

10* Were outcomes measured in the same way for treatment groups?

11* Were outcomes measured in a reliable way?

12* Was appropriate statistical analysis used?

13* Was the trial design appropriate, and any deviations from the standard RCT design (individual randomisation, parallel groups) accounted for in the conduct and analysis of the trial?

**Table S3:** Methodological quality of quasi-experimental study

Studies	Criteria									

	1*	2*	3*	4*	5*	6*	7*	8*	9*	Overall
McMullen and McMullen ( 44)	Y	Y	N	Y	Y	Y	Y	Y	Y	8/9 88%
Roy et al. ( 43)	Y	Y	N	N	Y	U	Y	Y	Y	6/9 66%
Yankey and Urmi ( 42)	Y	Y	Y	Y	Y	Y	Y	Y	Y	9/9 100%
Ndetei et al. ( 45)	Y	Y	Y	N	Y	U	Y	Y	Y	7/9 77%
Mohammadi and Poursaberi ( 40)	Y	Y	U	Y	Y	N	Y	Y	Y	7/9 77%
Eslami et al. ( 41)	Y	Y	Y	Y	Y	Y	Y	Y	Y	9/9 100%
McMahon and Hanrahan ( 46)	Y	Y	Y	Y	Y	Y	Y	U	Y	8/9 88%

Total	7/7 100%	7/7 100%	4/7 57%	5/7 71%	7/7 100%	4/7 57%	7/7 100%	6/7 85%	7/7 100%	

Notes: JBI methodological quality appraisal checklist to be scored as: Yes = Y; No = N; Unclear = U; Not applicable = NA;

1* Is it clear in the study what is the ‘cause’ and what is the ‘effect’ (i.e. there is no confusion about which variable comes first)?

2* Were the participants included in any comparisons similar?

3* Were the participants included in any comparisons receiving similar treatment/care, other than the exposure or intervention of interest?

4* Was there a control group?

5* Were there multiple measurements of the outcome both pre- and post-intervention/exposure?

6* Was follow up complete and if not, were differences between groups in terms of their follow up adequately described and analysed?

7* Were the outcomes of participants included in any comparisons measured in the same way?

8* Were outcomes measured in a reliable way?

9* Was appropriate statistical analysis used?

## Figures and Tables

**Table 1:** Study characteristics

Authors, year	Country	Design	Participants characteristics/Sample size	Setting	Trainer	Methodological quality
Mohammadzadeh et al., 2019 (29)	Malaysia	RCT	271 male and female adolescents (13 years old– 18 years old)	Orphanages	Researcher	High (85%)
Lee et al., 2020 (38)	Taiwan	RCT	2,522 students with age 10-year-old to 12-year-old	School	Teacher	Moderate (62%)
Jamali et al., 2016 (39)	Iran	Experimental (pre-post-tests) and control group	100 students, aged 13 years old –14 years old	School	Qualified trainers	Moderate (69%)
Yankey and Urmi, 2012 (42)	India	A quasi-experimental study	600 Tibetan adolescents aged 13 years old– 19 years old	School	Researcher	High (100%)
McMullen and McMullen, 2018 (44)	Uganda	Experimental study (pre-post-tests) and control group	620 students aged 13 years old –18 years old at the baseline and 170 students at post-intervention ‘at 1 year’ were participated	School	Teachers	High (88%)
Roy et al., 2016 (43)	India	Intervention study (pre-post-follow up)	42 adolescent boys, mean age (SD) 14.38 (1.05) years old	School	Researcher	Moderate (66%)
Ndetei et al., 2019 (45)	Kenya	Intervention (pre-post-follow up)	2,273 students at baseline, and only 1,075 complete the questionnaire for 9 months. Age from 11 years old to 18 years old	School	Trained teachers	Moderate (77%)
Mohammadi and Poursaberi, 2018 (40)	Iran	A quasi-experimental study	120 Iranian adolescent cancer patients, aged 9 years old–18 years old	Hospital	Clinical psychologist	Moderate (77%)
Eslami et al., 2016 (41)	Iran	A quasi-experimental study	126 female students, the mean age group was 16 years old	School	Researcher	High (100%)
McMahon and Stephanie, 2020 (46)	Australia	Experimental (pre-post-tests) and control group	40 students aged from 16 years old to 17 years old	School	Teacher	High (88%)

**Table 2:** Study instrument and findings

Authors, year	Instrument/Psychometric properties	Data collection period	Intervention characteristics	Finding
Mohammadzadeh et al., 2019 (29)	Validated Depression Anxiety Stress Scales (DASS-21), with Cronbach’s alpha coefficients for depression = 0.81, anxiety = 0.79 and stress = 0.81	Pre-intervention Immediately post-intervention 4 months post-intervention	20 activities were conducted by the researcher, twice weekly for 2 h to 2½ h per session in the Malay language	The mean scores of depressions, anxiety, stress was significantly decreased compared to the pre-test scores for depression (*F* = 33.80; *P* < 0.001; η^2^ = 0.11), for anxiety (*F* = 6.28; *P* = 0.01; η^2^ = 0.02), stress (*F* = 32.05; *P* < 0.001; η^2^ = 0.11)
Lee et al., 2020 (38)	Center for epidemiologic studies depression scale for children (CESDC), with Cronbach alpha, was 0.85	Post-intervention	27 class sessions were conducted for 45 min by the teacher	Life skills was associated with reduction of depressive symptoms among males but not females. Boys in the Life Skills group had significantly lower total CESDC scores and lower depressed affect scores (M = 10.49, SD = 7.47; M = 2.14, SD = 3.43, respectively) than those in the education as usual group (M = 11.64, SD = 9.14; M = 2.71, SD = 4.37, respectively)
Jamali et al., 2016 (39)	Validated stress questionnaire (based on Kettle personality scale), with Cronbach’s alpha for stress (α = 0.76)	pre-intervention post-intervention	Qualified trainers provided eight sessions (two sessions a week for 2 h) to the intervention group for 1 month	The mean scores of the stress factor in the intervention group (18.48) and control group (22.18) was statistically significant, *F* (2, 97) = 6.15, *P* < 0.001, η^2^ = 0.113
Yankey and Urmi, 2012 (42)	The Problem Questionnaire for stress, with reliability (Cronbach alpha = 0.83) and validity (from 0.18 to 0.45)	Pre-intervention Post-intervention after (2 weeks)	30 basic sessions and 15 additional sessions for students who were not able to comprehend life skills in one session. Follow up assessments were done 2 weeks post-intervention	Life skills have significantly contributed to reducing stress related to school, leisure and self among Tibetan adolescents. School stress for the experimental group was significantly lower (M = 20.84, SD = 4.92) as compared to the control group (M = 22.64, SD = 5.34) in the post-intervention scores
McMullen and McMullen, 2018 (44)	The African Youth Psychosocial Assessment Instrument (AYPA) for ‘depression/anxiety-like symptoms, with Cronbach’s alpha (α = 0.86)	Pre-intervention Post-intervention (after 1 year)	There were around 24 lessons conducted by teachers for 45 min–60 min	The intervention group had a significant reduction in internalising problems (depression/anxiety-like symptoms), *F* (1,167) = 11.14, *P* = 0.001, η^2^ = 0.063
Roy et al., 2016 (43)	Manipal Stress Questionnaire (MSQ), psychometric property was not documented	Pre-intervention 1-month post-intervention 3 months post-intervention	Validated 7 days sessions programme. The programme was conducted for 50 min–60 min	The mean stress scores among adolescents who underwent the intervention program reduced significantly from 133 to 116 after 1 month and to 117 after 3 months follow up (*P* < 0.05)
Ndetei et al., 2019 (45)	Youth self-report (YSR), with (Cronbach’s alpha = 0.82) and high test-retest reliably (*r* = 0.88)	Pre-intervention 9 months post-intervention	The training session was done at 8 h for 4 weeks with all schools	Life Skill intervention was significantly improving in the internalising YSR symptoms. There was an overall decrease in the internalising problems from 36.8% to 7.3%. AOR = 0.12; 95% CI: 0.09, 0.16. Better outcomes among girls than boys, rural region than urban, and in upper classes than in lower
Mohammadi and Poursaberi, 2018 (40)	The General Health Questionnaire (GHQ), with (Cronbach’s alpha = 0.90)	Pre-intervention Post-intervention	A clinical psychologist provided 13 training sessions for 45 min	The mean score of depressions, anxiety was decreased significantly after the training program, the anxiety score in the intervention group was M(SD) = 6.61 (2.62), compared to the control group M(SD)= 10.33 (2.37). While the depression score was 11.05 (2.84) for the intervention and 15.95 (2.33) for the control group
Eslami et al., 2016 (41)	Depression anxiety stress scales (DASS-21), with validity and reliability, were confirmed	Pre-intervention immediately post-intervention 2 months post-intervention	Eight sessions for 45 min were conducted by the researcher for 3 months	The results revealed a significant decrease in the level of anxiety and stress in the experimental group as compared to the control group after 2 months of the intervention (*P* < 0.001). However, there was no significant difference in the depression score in the intervention group immediately and 2 months post-intervention (*P* < 0.09)
McMahon and Stephanie, 2020 (46)	The Social Interaction Anxiety Scale (SIAS), with Cronbach’s alpha (α = 0.82)	Pre-test Post-test	10-session life skills programme with 2 h for 2 weeks was provided by the teacher	The result showed a significant decrease in social anxiety, Wilk’s Lamda = 0.84, *F* (1, 26) = 5.07; *P* = 0.03, partial *η*^2^ = 0.16 among the experimental group compared to the control group
